# Race, Ethnicity, and Sleep in US Children

**DOI:** 10.1001/jamanetworkopen.2024.49861

**Published:** 2024-12-10

**Authors:** Yijie Wang, Zhenqiang Zhao, Youchuan Zhang, Jinjin Yan, Meng-Run Zhang, Elizabeth Jelsma, Shadane Johnson, Heining Cham, Margarita Alegría, Tiffany Yip

**Affiliations:** 1 Department of Human Development and Family Studies, Michigan State University, East Lansing; 2 Department of Psychology, Fordham University, Bronx, New Jersey; 3 Disparities Research Unit, Massachusetts General Hospital, Boston, Massachusetts

## Abstract

**Importance:**

Although racial and ethnic disparities are well documented in children’s mean levels of sleep, particularly duration, evidence is mixed for sleep variability, an important sleep dimension of growing interest. Most research has also focused on comparisons of Black and White children, with limited attention to sleep variability disparities among other racially and ethnically minoritized groups such as Asian, Latinx, and multiracial children.

**Objective:**

To investigate racial and ethnic disparities in children’s mean levels of sleep and variability of sleep across multiple dimensions and diverse racial and ethnic groups using actigraphy data.

**Design, Setting, and Participants:**

This cross-sectional study used data from the Adolescent Brain Cognitive Development (ABCD) study, a national cohort study, from 21 study sites across the US. ABCD participants were recruited at baseline (2016-2018) using a multistage, stratified, probability sampling method. The current study used a subsample with reliable actigraphy data collected at 2-year follow-up (2018-2020). Data analysis occurred from July 2023 to October 2024.

**Exposures:**

Parent-reported race and ethnicity at baseline. Sociodemographic, health, and contextual covariates of sleep were also included.

**Main Outcomes and Measures:**

Actigraphy-assessed mean levels of sleep and sleep variability across multiple dimensions (duration, bedtime, risetime, efficiency, and latency) over 3 weeks.

**Results:**

The analytic sample included 3868 children (mean [SD] age, 11.50 [0.67] years; 1913 female [49.5%]), of whom 104 (2.7%) were Asian, 347 (9.0%) were Black or African American, 801 (20.7%) were Latinx, 356 (9.2%) were multiracial, and 2260 (58.4%) were White. Asian, Black, Latinx, and multiracial children exhibited shorter sleep duration and later bedtime than White children. Importantly, compared with White children, bedtime variability was greater among Asian (β = 0.04; 95% CI, 0.01 to 0.07; *P* = .02), Black (β = 0.11 95% CI, 0.08 to 0.15; *P* < .001), Latinx (β = 0.08; 95% CI, 0.05 to 0.12; *P* < .001), and multiracial children (β = 0.08; 95% CI, 0.05 to 0.11; *P* < .001). Similarly, risetime variability was greater among Asian (β = 0.04; 95% CI, 0.01 to 0.07; *P* = .01), Black (β = 0.08; 95% CI, 0.04 to 0.12; *P* < .001), and Latinx (β = 0.06; 95% CI, 0.02 to 0.10; *P* < .01) children in comparison with White children. Black children exhibited the most profound disparities across mean levels (duration and bedtime) and variability of sleep (duration, bedtime, risetime, and efficiency) than other groups. Asian and multiracial children also exhibited some disparities sleep duration, efficiency, and efficiency variability, when compared with Latinx children.

**Conclusions and Relevance:**

In this cross-sectional study of children’s sleep disparities, racially and ethnically minoritized children exhibited disparities in mean levels and variability of sleep compared with their White peers. These findings suggest that policies and practices should target multiple sleep dimensions among diverse racial and ethnic groups to promote equitable pediatric sleep health.

## Key Points

**Question:**

Are measures of mean levels of sleep and sleep variability among children different by race and ethnicity?

**Findings:**

In this cross-sectional study of 3868 children that used actigraphy sleep data from the Adolescent Brain Cognitive Development study, Asian, Black, Latinx, and multiracial children consistently exhibited shorter sleep duration and later bedtime, as well as greater variability in bedtime and risetime, than their non-Hispanic White peers.

**Meaning:**

These findings suggest that to effectively promote sleep equity, policies and practices should address not only mean sleep levels but also variability in sleep patterns among children from diverse racial and ethnic groups.

## Teaser

This cross-sectional study analyzes differences in sleep levels and sleep variability among racially and ethnically diverse children in the US.

## Introduction

Sleep is essential for pediatric health across domains.^1,2^ Unfortunately, not all children have an equal opportunity for adequate and quality sleep. A growing literature shows that children from racially and ethnically minoritized groups, especially those from Black families, tend to have shorter sleep duration,^3,4,5,6,7,8,9,10,11^ and, to some extent, later bedtime^5,12^ and poorer sleep quality^11,13^ than their non-Hispanic White peers.^1,14,15^ These racial disparities in sleep duration have also been documented by recent national studies^16,17^ using objective, actigraphy sleep data that are free of recall and reporting bias.^18^ Theoretical and empirical work attribute these disparities to structural and interpersonal challenges associated with children’s racially and ethnically minoritized identities, as well as cultural differences in the beliefs and practices related to sleep.^14,19^

Beyond mean levels of sleep, research increasingly recognizes the importance of sleep variability (fluctuations from one day to another),^20,21^ which has been associated with children’s health and development across domains above and beyond sleep duration.^22^ Variability in sleep can disrupt its alignment with the body’s circadian rhythm, which is essential for optimal daily functioning and healthy brain development.^20^ Theoretical work suggests that factors (eg, structural and interpersonal racism or cultural differences) that contribute to disparities in mean levels of sleep also matter for sleep variability.^14,19^ However, empirical findings are inconsistent. Some studies found greater variability in sleep duration and bedtime (measured via actigraphy) among Black children, or from combined racially and ethnically minoritized groups primarily composed of Black children, in comparison with their White peers.^9,12,23,24^ However, other studies observed nonsignificant differences (again primarily between Black and White children) using actigraphy method^13,25,26^ or self-reports.^27^

There is also limited attention to groups beyond Black children. Two studies examined disparities in sleep duration variability among Latinx and Asian children, again yielding mixed findings; one study showed that Asian (but not Latinx) youths exhibited lower variability based on actigraphy than White youths,^28^ while the other study observed nonsignificant differences based on self-reports.^29^ Little to no research has examined disparities in sleep variability for multiracial children, who also face stress associated with their racially and ethnically minoritized identities that may influence sleep.^30^ This lack of research is partly due to the use of small community samples that limit comparisons between diverse groups.^31^ The mixed findings may also stem from small community samples with limited power to detect group differences, as well as sleep assessment method (eg, studies using self-reports yielded more null findings^27,29^).

Leveraging actigraphy sleep data collected by the national Adolescent Brain Cognitive Development (ABCD) study, the current study contributes to pediatric sleep science by investigating sleep disparities in both mean levels and variability of sleep across multiple dimensions (duration, bedtime, risetime, efficiency, and latency) among children from diverse racial and ethnic groups (Asian, Black, Latinx, multiracial, and White). The national sample ensured representations of more diverse racially and ethnically minoritized groups, allowing for the generalization of study findings to a broader population. We accounted for other known sources of variation in children’s sleep, including sociodemographic, health, and contextual factors.^3,4,9^ For example, children from families with lower socioeconomic status (SES) and more disadvantaged neighborhoods tend to have shorter sleep.^9,16^ Potential variations by weekday vs weekends^3^ were also explored. While we expected to identify significant racial and ethnic disparities in mean levels of sleep, no hypotheses were posed for sleep variability indicators, given the mixed findings of previous research.

## Method

### Participants

In this cross-sectional study, data were drawn from the 5.0 data release of the ABCD study, an ongoing longitudinal cohort study examining children’s brain development and health outcomes from late childhood to adolescence in the US. In the baseline year (2016-2018), 11 876 children aged 9 to 11 years were recruited from 21 study sites across the country using stratified probability sampling. Written informed consent from the primary caregiver and assent from the children were obtained. A centralized institutional review board approval was obtained from the University of California, San Diego, and study sites also received approval from their local institutional review boards. This secondary data analysis was determined as exempt from additional institutional review board review by Michigan State University.

At 2-year follow-up (2018-2020), a 3-week actigraphy sleep assessment was administered among 5901 children. The analytic sample was selected in the following steps. First, participants whose actigraphy assessment dates were missing or outside of the 3-week protocol (76 participants) and who had less than 6 days of actigraphy data to assess sleep reliably^32^ (1024 participants) were excluded. The missingness of the original sleep data (18.5%) was associated with study variables (race and ethnicity and covariates) in small effect sizes (median [range] *r*, 0.04 [0.00-0.17]; see eTable 1 in Supplement 1), suggesting that the data were missing completely at random based on methodological recommendations.^33^ To address potential nonindependence within families, 1 child was randomly selected from each participating family (848 excluded). Finally, participants with missing race and ethnicity (49 participants) or from groups with limited sample sizes (11 single-race American Indian and Alaska Native participants, 2 Pacific Islander participants, and 23 participants in the other race or ethnicity category) were excluded. Because the ABCD study does not provide weights for the actigraphy subsample, we compared the analytic sample with the full ABCD sample. This study followed the Strengthening the Reporting of Observational Studies in Epidemiology (STROBE) reporting guideline.

### Measures

#### Actigraphy Sleep

Children’s sleep was assessed at 2-year follow-up using Fitbit Charge 2 actigraph watches, which performed comparably to other reliable, research-based actigraphs when validated against the benchmarkpolysomnography (see eTable 2 in Supplement 1 for validation details).^34,35^ Sleep intervals were identified by a proprietary algorithm incorporating motion and heart rate data in 1-minute epochs. Informed by previous research on sleep disparities,^14,15^ we examined dimensions of duration, bedtime, risetime, latency, and efficiency. Sleep duration was computed by the ABCD staff, assessing the total time (in hours) spent asleep across various stages (light, deep, and rapid eye movement). Bedtime captured the first minute a participant was identified as being in bed (but not necessarily asleep). The raw bedtime data were converted to numeric values (in hours) and centered by noon (eg, 10:15 PM is denoted as 10.25; 1:30 AM as 13.50), so that bedtime before and after midnight was assessed continuously, with higher scores indicating later bedtime. Sleep latency captured the difference (in minutes) between bedtime and sleep onset time. Risetime was the first minute a participant was identified as being out of bed (but may be awake before that). The raw risetime data were converted to numeric values and centered by midnight (eg, 7:45 AM is denoted as 7.75) to facilitate interpretation. Higher scores indicated later risetime. Sleep efficiency was calculated as the proportion of sleep duration divided by total time spent in bed (0%-100%).

For each sleep dimension, we calculated its mean and variability (ie, SD)^36,37^ over the 3-week study period for each participant. The SD represents the average deviation between a participant’s daily sleep and their mean. Because the SD values were positively skewed, we used raw variability variables in descriptive statistics but log-transformed them into normally distributed variables for analysis. We also calculated sleep mean and variability separately for weekdays vs weekends for participants with at least 20% of valid data (3 weekdays and 2 weekends).

Supporting construct validity, recent studies have associated actigraphy sleep duration, quality, and variability with internalizing symptoms,^38^ brain development,^39^ and obesity^40^ in the ABCD study. We also observed associations of multiple sleep dimensions (mean and variability) with children’s cognitive functions, school outcomes, and psychopathology symptoms (see eTable 3 in Supplement 1).

#### Race and Ethnicity

Parents reported children’s race and ethnicity at baseline. Participants were coded as Latinx if their parents answered yes to the question, “Do you consider the child Hispanic/Latino/Latina?” Based on parent reports to the question, “What race do you consider the child to be?” the remaining participants were categorized into 1 of 6 groups: White, Black or African American, American Indian or Alaska Native, Asian (defined as Asian Indian, Chinese, Filipino, Japanese, Korean, Vietnamese, and other Asian [ie, any Asian ethnicity not otherwise specified]), Pacific Islander (Native Hawaiian, Guamanian, Samoan, or Other Pacific Islander), and other (defined as any race not otherwise specified). Children were then coded as multiracial if parents selected more than 1 racial group. Due to limited sample sizes, single-race American Indian or Alaska Native participants, Pacific Islander participants, and participants in the other race category were excluded from the study, as stated previously. Racial and ethnic groups were dummy coded, with the reference group rotated to enable all possible comparisons.

#### Covariates

Measurement details are presented in eTable 4 in Supplement 1. For sociodemographics, parents reported their children’s age, sex assigned at birth, generational status, and family structure at baseline. Family SES was assessed as a latent factor indicated by parent-reported education, employment status, and economic hardship. Children’s sexual orientation was identified based on self-reports from baseline to 2-year follow-up. For health factors, children’s body mass index (calculated as weight in kilograms divided by height in meters squared) was collected by the ABCD team at 2-year follow-up and standardized as *z* scores based on the Centers for Disease Control and Prevention 2000 Growth Chart.^41^ Children also reported their weekly caffeine intake over the past 6 months (5 items), physical activity in the past week (1 item), and bedtime screen usage in the past week (10 items) at 2-year follow-up. Regarding contextual factors, the ABCD team assessed neighborhood deprivation using an 18-item Area Deprivation Index^42^ based on baseline geocode data. This index captured education, employment, income, housing, poverty rate, and infrastructure in one’s neighborhood. Children also reported on the time when their usual school schedule starts at 2-year follow-up.

### Statistical Analysis

Path analyses were conducted in Mplus version 8.10 (Muthén et al).^43^ No missing data were present in sleep or race and ethnicity variables as stated previously. Missing data in other study variables (0%-10%) were handled by the full information maximum likelihood method. Nonindependence by study sites was addressed by the cluster and type = complex feature in Mplus.

Primary analyses examined racial and ethnic disparities in sleep across all study days. Separate models were fitted for sleep mean and variability. In each model, multiple sleep dimensions (duration, bedtime, risetime, latency, and efficiency) were simultaneously estimated by dummy-coded race and ethnicity variables (with the reference group rotated to obtain all possible comparisons). To account for multiple comparisons across racial and ethnic groups, the false discovery rate was adjusted using the Benjamini-Hochberg procedure,^44^ a preferred approach over setting an arbitrary, more conservative α level (eg, *P* < .01). Covariates were included as associative factors of sleep in all models. Supplementary analyses also investigated raw racial and ethnic differences without covariates, and how the differences changed when introducing the 3 sets of covariates (sociodemographic, health, and contextual) one at a time.

To examine weekday-weekend variations, for each sleep indicator, we compared a model that freely estimated the effect size of each race and ethnicity variable on weekday and weekend sleep with a model that constrained the effect size to be equal. Model comparisons used the Satorra-Bentler scaled χ^2^ test. The threshold for statistical significance was a 2-sided *P* < .05. Data analysis was conducted from July 2023 to October 2024.

## Results

The final analytic sample included 3868 children (mean [SD] age, 11.50 [0.67] years; 1913 female [49.5%]), of whom 104 (2.7%) were Asian, 347 (9.0%) were Black or African American, 801 (20.7%) were Latinx, 356 (9.2%) were multiracial, and 2260 (58.4%) were White (Table 1). Table 2 presents descriptive statistics for sleep across all study days (see weekday and weekend sleep in eTable 5 in Supplement 1). Bivariate correlations were generally less than 0.70,^45^ suggesting that the variables each captured a unique aspect of sleep. The only exception was for latency mean and variability (*r* = 0.82; *P* < .001), suggesting potential redundancy between the 2 variables.

**Table 1.** Participant Characteristics^a^

Variable	Sample, No. (%)		Sample difference		
	ABCD (N = 11 876)	Analytic (N = 3868)	*F* (df)	*t* (df)	*P* value
Race and ethnicity					
Asian	253 (2.2)	104 (2.7)	41.31 (7)	NA	<.001
Black or African American	1765 (15.1)	347 (9.0)			
Latinx	2411 (20.6)	801 (20.7)			
Multiracial	1080 (9.2)	356 (9.2)			
American Indian or Alaska Native	38 (0.3)	NA			
Pacific Islander	13 (0.1)	NA			
White	6094 (52.0)	2260 (58.4)			
Other	57 (0.5)	NA			
Do not know	5 (0.05)	NA			
Other race (not specified)	10 (0.1)	NA			
Refuse to answer	11 (0.1)	NA			
Missing	31 (.25)	NA			
Age, mean (SD), y	9.48 (0.51)^b^	11.50 (0.67)^c^	NA	0.53 (6591.5)	.59
Sex					
Female	5680 (47.8)	1913 (49.5)	3.05 (1)	NA	.08
Male	6196 (52.2)	1955 (50.5)			
Sexual orientation (gay or bisexual)					
No	10 281 (90.8)	3435 (89.7)	3.57 (1)	NA	.06
Yes	1046 (9.2)	394 (10.3)			
Generational status					
Child or parent foreign born	2624 (22.4)	921 (24.1)	4.92 (1)	NA	.03
Child and both parents US born	9097 (77.6)	2894 (75.9)			
Parental educational level					
<High school diploma	564 (4.8)	108 (2.8)	111.64 (6)	NA	<.001
High school diploma or GED	1101 (9.4)	236 (6.1)			
Some college	1480 (12.6)	415 (10.8)			
Associate’s degree	1559 (13.3)	452 (11.8)			
Bachelor’s degree	3001 (25.6)	1076 (28.0)			
Master’s degree	2801 (23.9)	1090 (28.4)			
Doctoral degree	1237 (10.5)	466 (12.1)			
Parent employment status					
Not working	857 (7.8)	198 (5.3)	42.17 (2)	NA	<.001
Part-time working	572 (5.2)	163 (4.3)			
Full-time working	9596 (87.0)	3389 (90.4)			
Family economic hardship score, mean (SD)	0.45 (1.10)	0.38 (1.02)	NA	−4.10 (7577.0)	<.001
2-Parent family					
No	3102 (26.3)	789 (20.5)	NA	22.50 (1.0)	<.001
Yes	8678 (73.7)	3061 (79.5)			
BMI, mean (SD), *z* score	0.48 (1.15)	0.44 (1.13)	NA	−1.68 (6883.0)	.09
Weekly caffeine use, mean (SD), No. of drinks	1.95 (5.07)	1.91 (5.25)	NA	0.38 (6559.0)	.70
Physical activity, mean (SD), h/wk	3.77 (2.16)	3.91 (2.09)	NA	3.53 (6969.0)	<.001
Neighborhood deprivation score, mean (SD)	40.04 (26.97)	34.90 (24.75)	NA	−3.68 (2879.8)	<.001
School start time, mean (SD)	8.30 (0.98)	8.26 (0.92)	NA	−2.31 (7276.2)	.02
Region					
Northeast	2005 (16.9)	792 (20.5)	58.48 (3)	NA	<.001
Mideast	2419 (20.4)	650 (16.8)			
South	3364 (28.3)	975 (25.2)			
West	4084 (34.4)	1450 (37.5)			

Abbreviations: ABCD, Adolescent Brain Cognitive Development study; BMI, body mass index; GED, general educational development; NA, not applicable.

^a^ Binary covariates were coded as follows in analysis: sex assigned at birth (0 = male; 1 = female), generational status (0 = the child or at least 1 of the parents was foreign born; 1 = the child and both parents were US born), 2-parent household (0 = no; 1 = yes), sexual orientation of gay or bisexual at any wave (1 = yes; 0 = no). Family socioeconomic status was assessed as a latent factor indicated by parent-reported education (λ = 0.71), employment status (λ = 0.48), and economic hardship (λ = −0.39).

^b^ At baseline.

^c^ At 2-year follow-up.

**Table 2.** Descriptive Statistics of Multiple Sleep Dimensions Across All Possible Days

Variable	Outcome, mean (SD) [90% CI]	Bivariate correlations																	
		Mean levels								Variability									
		Bedtime		Rise time		Efficiency		Latency		Duration		Bedtime		Rise time		Efficiency		Latency	
		*r*	*P* value	*r*	*P* value	*r*	*P* value	*r*	*P* value	*r*	*P* value	*r*	*P* value	*r*	*P* value	*r*	*P* value	*r*	*P* value
**Sleep level**																			
Duration, h	7.46 (0.64) [6.33-8.38]	−0.41	<.001	0.16	<.001	0.14	<.001	0.12	<.001	−0.31	<.001	−0.33	<.001	−0.24	<.001	−0.26	<.001	0.13	<.001
Bedtime, h	10.96 (1.19) [9.35-13.26]	NA	NA	0.03	.11	0.02	.34	0.02	.32	0.39	<.001	0.45	<.001	0.54	<.001	0.15	<.001	−0.01	.47
Rise time, h	7.13 (1.43) [5.38-8.82]	NA	NA	NA	NA	0.07	<.001	0.02	.14	−0.14	<.001	−0.17	<.001	−0.36	<.001	−0.12	<.001	0.01	.47
Efficiency score (0-1)	0.87 (0.02) [0.83-0.90]	NA	NA	NA	NA	NA	NA	0.03	.04	−0.16	<.001	−0.12	<.001	−0.08	<.001	−0.56	<.001	0.05	.002
Latency, min	6.94 (4.80) [1.83-16.04]	NA	NA	NA	NA	NA	NA	NA	NA	0<.001	.92	0.04	.02	0.01	.60	0.03	.05	0.82	<.001
**Variability** ^a^																			
Duration, h	1.08 (0.50) [0.47-2.04]	NA	NA	NA	NA	NA	NA	NA	NA	NA	NA	0.60	<.001	0.62	<.001	0.36	<.001	0.01	.36
Bedtime, h	1.17 (0.77) [0.41-2.62]	NA	NA	NA	NA	NA	NA	NA	NA	NA	NA	NA	NA	0.55	<.001	0.31	<.001	0.04	.02
Risetime, h	1.61 (1.90) [0.40-6.43]	NA	NA	NA	NA	NA	NA	NA	NA	NA	NA	NA	NA	NA	NA	0.23	<.001	0.01	.65
Efficiency (0-1)	0.03 (0.03) [0.02-0.08]	NA	NA	NA	NA	NA	NA	NA	NA	NA	NA	NA	NA	NA	NA	NA	NA	0.03	.12
Latency, min	8.59 (7.45) [1.85-23.25]	NA	NA	NA	NA	NA	NA	NA	NA	NA	NA	NA	NA	NA	NA	NA	NA	NA	NA

Abbreviation: NA, not applicable.

^a^ Raw variability scores (ie, SD of each participant’s sleep data over 3 weeks) were used to estimate descriptive statistics. We then log-transformed the raw variability scores when estimating correlations.

### Racial and Ethnic Disparities in Sleep

#### Mean Levels

As shown in Table 3 (see visualization in Figure A-E), compared with White children, sleep duration was significantly shorter among Asian children (β = −0.08; 95% CI, −0.11 to −0.04; *P* < .001), Black children (β = −0.18; 95% CI, −0.22 to −0.14; *P* < .001), Latinx children (β = −0.06; 95% CI, −0.10 to −0.03; *P* = .002), and multiracial children (β = −0.06; 95% CI, −0.09 to −0.03; *P* < .001). Similarly, bedtime was significantly later among Asian children (β = 0.06; 95% CI, 0.03 to 0.09; *P* < .001), Black children (β = 0.13; 95% CI, 0.09 to 0.17; *P* < .001), Latinx children (β = 0.10; 95% CI, 0.06 to 0.13; *P* < .001), and multiracial children (β = 0.05; 95% CI, 0.02 to 0.09; *P* = .003) in comparison with White children. Latinx children had higher sleep efficiency than White children (β = 0.07; 95% CI, 0.03 to 0.11; *P* = .01).

**Table 3.** Standardized Coefficient Estimates for Racial and Ethnic Differences in Multiple Sleep Dimensions (Mean Levels)^a^

Variable	Duration, β (95% CI)	*P* value	Bedtime, β (95% CI)	*P* value	Risetime, β (95% CI)	*P* value	Efficiency, β (95% CI)	*P* value	Latency, β (95% CI)	*P* value
Race and ethnicity by reference group										
White as reference group										
Asian	−0.08 (−0.11 to −0.04)	<.001	0.06 (0.03 to 0.09)	<.001	0.00 (−0.04 to 0.03)	>.99	−0.02 (−0.05 to 0.01)	.05	−0.01 (−0.04 to 0.02)	.40
Black	−0.18 (−0.22 to −0.14)	<.001	0.13 (0.09 to 0.17)	<.001	−0.02 (−0.07 to 0.02)	.68	0.02 (−0.02 to 0.06)	.50	0.04 (0.00 to 0.08)	.23
Latinx	−0.06 (−0.10 to −0.03)	.002	0.10 (0.06 to 0.13)	<.001	0.04 (0.00 to 0.08)	.16	0.07 (0.03 to 0.11)	.01	0.01 (−0.04 to 0.05)	.25
Multiracial	−0.06 (−0.09 to −0.03)	<.001	0.05 (0.02 to 0.09)	.003	−0.01 (−0.04 to 0.03)	.89	−0.02 (−0.06 to 0.01)	.31	0.02 (−0.01 to 0.06)	.48
Black as reference group										
Asian	0.02 (−0.02 to 0.06)	.26	−0.01 (−0.05 to 0.03)	.52	0.01 (−0.03 to 0.05)	.92	−0.03 (−0.07 to 0.01)	.72	−0.04 (−0.07 to 0.00)	.58
Latinx	0.19 (0.13 to 0.25)	<.001	−0.09 (−0.15 to −0.03)	.01	0.07 (0.01 to 0.14)	.28	0.04 (−0.02 to 0.10)	.30	−0.05 (−0.11 to 0.01)	.25
Multiracial	0.12 (0.08 to 0.17)	<.001	−0.08 (−0.13 to −0.03)	.004	0.02 (−0.04 to 0.07)	.95	−0.04 (−0.09 to 0.01)	.27	−0.02 (−0.07 to 0.03)	.50
Latinx as reference group										
Asian	−0.05 (−0.09 to −0.02)	.003	0.02 (−0.01 to 0.05)	.19	−0.02 (−0.06 to 0.02)	.74	−0.05 (−0.08 to −0.02)	.01	−0.02 (−0.05 to 0.01)	.58
Multiracial	−0.01 (−0.05 to 0.02)	.45	−0.02 (−0.05 to 0.02)	.41	−0.04 (−0.08 to 0.00)	.19	−0.07 (−0.11 to −0.03)	<.001	0.02 (−0.02 to 0.06)	.50
Multiracial as reference group										
Asian	−0.05 (−0.08 to −0.01)	.02	0.03 (0.00 to 0.06)	.09	0.00 (−0.04 to 0.04)	.95	−0.01 (−0.04 to 0.03)	.72	−0.03 (−0.06 to 0.01)	.95
Covariate										
Age	−0.13 (−0.16 to −0.10)	<.001	0.16 (0.13 to 0.19)	<.001	0.03 (0.00 to 0.06)	.09	−0.01 (−0.04 to 0.03)	.72	0.02 (−0.01 to 0.05)	.24
Female	0.13 (0.10 to 0.16)	<.001	0.01 (−0.03 to 0.04)	.74	0.06 (0.03 to 0.09)	<.001	0.13 (0.10 to 0.17)	<.001	0.07 (0.03 to 0.10)	<.001
Sexual orientation	−0.04 (−0.07 to −0.01)	.02	0.05 (0.02 to 0.09)	.001	−0.01 (−0.04 to 0.03)	.75	0.01 (−0.02 to 0.04)	.55	−0.01 (−0.04 to 0.02)	.60
Generational status	−0.04 (−0.08 to −0.01)	.01	0.03 (0.00 to 0.06)	.08	−0.01 (−0.05 to 0.02)	.48	−0.01 (−0.05 to 0.03)	.61	−0.02 (−0.06 to 0.02)	.28
SES	0.01 (−0.03 to 0.04)	.77	−0.07 (−0.11 to −0.04)	<.001	0.04 (0.00 to 0.08)	.09	0.01 (−0.03 to 0.05)	.51	0.004 (−0.04 to 0.04)	.83
BMI	−0.09 (−0.12 to −0.06)	<.001	0.03 (0.00 to 0.06)	.07	−0.05 (−0.09 to −0.02)	.003	0.02 (−0.02 to 0.05)	.33	−0.05 (−0.09 to −0.02)	.004
Caffeine use	−0.02 (−0.05 to 0.01)	.23	0.04 (0.01 to 0.08)	.01	0.00 (−0.04 to 0.04)	.96	−0.02 (−0.05 to 0.02)	.36	0.00 (−0.03 to 0.04)	.91
Physical activity	0.02 (−0.01 to 0.05)	.31	−0.04 (−0.07 to −0.01)	.02	0.03 (−0.003 to 0.06)	.08	0.02 (−0.01 to 0.05)	.28	−0.01 (−0.05 to 0.02)	.39
Screen use	−0.13 (−0.16 to −0.09)	<.001	0.14 (0.11 to 0.18)	<.001	−0.04 (−0.08 to 0.00)	.05	−0.01 (−0.05 to 0.02)	.55	0.03 (0.00 to 0.07)	.04
2-Parent family	0.08 (0.04 to 0.11)	<.001	−0.04 (−0.07 to 0.00)	.03	0.06 (0.02 to 0.10)	.002	−0.01 (−0.05 to 0.02)	.53	0.01 (−0.03 to 0.04)	.72
Neighborhood deprivation score	−0.09 (−0.13 to −0.05)	<.001	0.12 (0.08 to 0.15)	<.001	−0.03 (−0.07 to 0.01)	.12	−0.04 (−0.08 to 0.01)	.09	0.03 (−0.01 to 0.07)	.10
School start time	0.07 (0.04 to 0.10)	<.001	0.10 (0.07 to 0.13)	<.001	0.14 (0.11 to 0.17)	<.001	−0.01 (−0.05 to 0.02)	.39	0.00 (−0.03 to 0.03)	.79

Abbreviations: BMI, body mass index; SES socioeconomic status.

^a^ False discovery rate (significance level) was adjusted using the Benjamini-Hochberg procedure. The actual disparities calculated by unstandardized coefficient estimates are reported in eTable 8 in Supplement 1.

**Figure.**
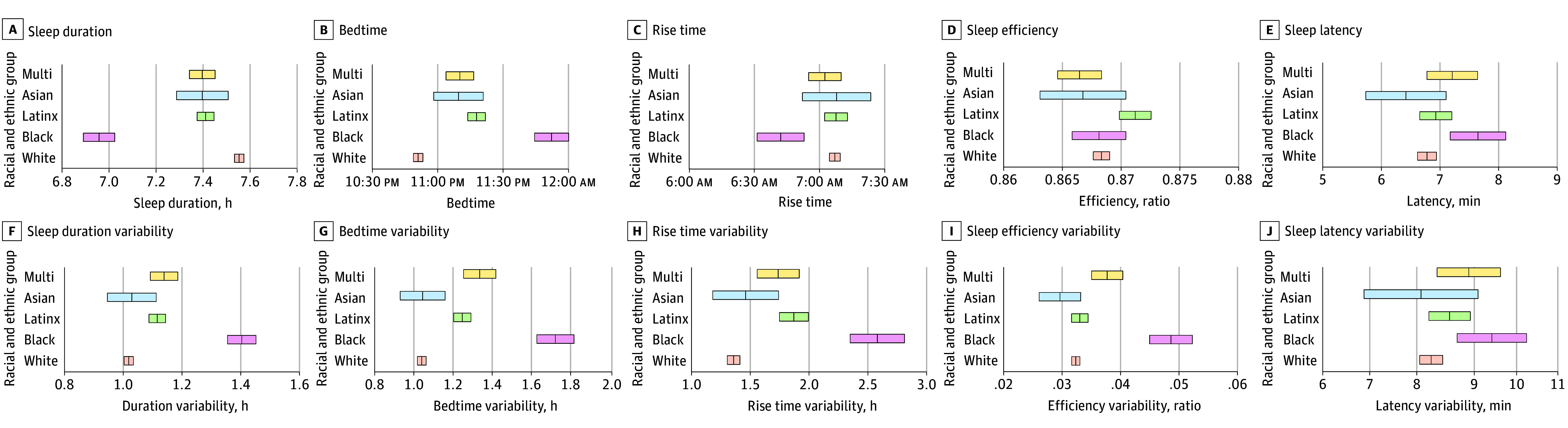
Sleep Levels and Variability of Sleep by Race and Ethnicity The line within each bar represents the mean, with the bandwidth of each bar indicating the 95% CI of the estimate (ie, sleep levels or sleep variability). Significant racial and ethnic differences were observed in the primary analysis for sleep duration (A), bedtime (B), sleep efficiency (D), sleep duration variability (F), bedtime variability (G), rise time variability (H), and sleep efficiency variability (I). Multi indicates multiracial.

Significant differences also emerged among racially and ethnically minoritized groups. Compared with Black children, Latinx and multiracial children had longer sleep duration (Latinx: β = 0.19; 95% CI, 0.13 to 0.25; *P* < .001; multiracial: β = 0.12; 95% CI, 0.08 to 0.17; *P* < .001) and earlier bedtime (Latinx: β = −0.09; 95% CI, −0.15 to −0.03; *P* = .01; multiracial: β = −0.08; 95% CI, −0.13 to −0.03; *P* = .004). Asian children had shorter sleep duration than Latinx children (β = −0.05; 95% CI, −0.09 to −0.02; *P* = .003) and multiracial children (β = −0.05; 95% CI, −0.08 to −0.01; *P* = .02). Moreover, Asian children (β = −0.05; 95% CI, −0.08 to −0.02; *P* = .01) and multiracial children (β = −0.07; 95% CI, −0.11 to −0.03; *P* < .001) showed lower efficiency than Latinx children.

#### Variability

As shown in Table 4 (see visualization in Figure F-J), compared with White children, there was greater variability in bedtime among Asian children (β = 0.04; 95% CI, 0.01 to 0.07; *P* = .02), Black children (β = 0.11; 95% CI, 0.08 to 0.15; *P* < .001), Latinx children (β = 0.08; 95% CI, 0.05 to 0.12; *P* < .001), and multiracial children (β = 0.08; 95% CI, 0.05 to 0.11; *P* < .001). Similarly, there was consistently greater variability in rise time among Asian children (β = 0.04; 95% CI, 0.01 to 0.07; *P* = .01), Black children (β = 0.08; 95% CI, 0.04 to 0.12; *P* < .001), and Latinx children (β = 0.06; 95% CI, 0.02 to 0.10; *P* = .01) in comparison with White children. Black and multiracial children also showed greater variability in sleep duration (Black: β = 0.09; 95% CI, 0.05 to 0.12; *P* < .001; multiracial: β = 0.04; 95% CI, 0.01 to 0.07; *P* = .04) and efficiency (Black: β = 0.11; 95% CI, 0.07 to 0.16; *P* < .001; multiracial: β = 0.04; 95% CI, 0.01 to 0.08; *P* = .03) than their White peers.

**Table 4.** Standardized Coefficient Estimates for Racial and Ethnic Differences in Multiple Sleep Dimensions (Variability)^a^

Variable	Duration, β (95% CI)	*P* value	Bedtime, β (95% CI)	*P* value	Risetime, β (95% CI)	*P* value	Efficiency, β (95% CI)	*P* value	Latency, β (95% CI)	*P* value
White as reference group										
Asian	0.03 (−0.01 to 0.06)	.18	0.04 (0.01 to 0.07)	.02	0.04 (0.01 to 0.07)	.01	0.00 (−0.03 to 0.02)	.77	−0.01 (−0.04 to 0.02)	.74
Black	0.09 (0.05 to 0.12)	<.001	0.11 (0.08 to 0.15)	<.001	0.08 (0.04 to 0.12)	<.001	0.11 (0.07 to 0.16)	<.001	0.02 (−0.02 to 0.06)	.78
Latinx	0.03 (−0.01 to 0.07)	.19	0.08 (0.05 to 0.12)	<.001	0.06 (0.02 to 0.10)	.01	−0.02 (−0.06 to 0.02)	.34	0.00 (−0.04 to 0.04)	.97
Multiracial	0.04 (0.01 to 0.07)	.04	0.08 (0.05 to 0.11)	<.001	0.04 (0.00 to 0.07)	.07	0.04 (0.01 to 0.08)	.03	0.02 (−0.02 to 0.05)	.99
Black as reference group										
Asian	−0.02 (−0.06 to 0.02)	.38	−0.03 (−0.06 to 0.01)	.24	0.00 (−0.04 to 0.03)	.96	−0.07 (−0.10 to −0.03)	<.001	−0.02 (−0.06 to 0.02)	.98
Latinx	−0.09 (−0.15 to −0.04)	<.001	−0.08 (−0.13 to −0.03)	.008	−0.05 (−0.11 to 0.01)	.12	−0.18 (−0.25 to −0.11)	<.001	−0.02 (−0.08 to 0.03)	.82
Multiracial	−0.05 (−0.09 to −0.01)	.06	−0.03 (−0.08 to 0.01)	.21	−0.04 (−0.09 to 0.00)	.12	−0.07 (−0.12 to −0.01)	.03	0.00 (−0.05 to 0.05)	.93
Latinx as reference group										
Asian	0.02 (−0.02 to 0.05)	.36	0.01 (−0.02 to 0.04)	.66	0.02 (−0.01 to 0.05)	.24	0.01 (−0.02 to 0.03)	.83	−0.01 (−0.05 to 0.02)	.78
Multiracial	0.02 (−0.02 to 0.05)	.42	0.03 (−0.01 to 0.06)	.19	−0.01 (−0.04 to 0.03)	.80	0.06 (0.02 to 0.10)	.01	0.02 (−0.02 to 0.05)	.72
Multiracial as reference group										
Asian	0.01 (−0.03 to 0.04)	.71	−0.01 (−0.04 to 0.02)	.71	0.02 (−0.01 to 0.06)	.21	−0.03 (−0.06 to 0.00)	.11	−0.02 (−0.06 to 0.02)	.99
Covariates										
Age	0.07 (0.04 to 0.10)	<.001	0.07 (0.04 to 0.10)	<.001	0.08 (0.05 to 0.11)	<.001	0.01 (−0.02 to 0.05)	.38	−0.01 (−0.04 to 0.02)	.43
Female	0.04 (0.01 to 0.07)	.005	0.01 (−0.02 to 0.04)	.43	0.05 (0.02 to 0.08)	.002	−0.03 (−0.06 to 0)	.05	0.07 (0.04 to 0.10)	<.001
Sexual orientation	0.06 (0.03 to 0.09)	<.001	0.07 (0.04 to 0.10)	<.001	0.05 (0.02 to 0.08)	.002	0.01 (−0.02 to 0.05)	.50	−0.02 (−0.05 to 0.01)	.23
Generational status	0.05 (0.02 to 0.09)	.00	0.07 (0.03 to 0.10)	<.001	0.04 (0.01 to 0.08)	.02	0.05 (0.01 to 0.09)	.009	−0.02 (−0.06 to 0.02)	.37
SES	−0.10 (−0.14 to −0.07)	<.001	−0.10 (−0.14 to −0.07)	<.001	−0.14 (−0.17 to −0.10)	<.001	−0.03 (−0.07 to 0.01)	.15	0.00 (−0.04 to 0.04)	.87
BMI	0.03 (0.00 to 0.06)	.09	0.04 (0.01 to 0.07)	.01	0.05 (0.02 to 0.08)	.003	0.01 (−0.03 to 0.04)	.67	−0.04 (−0.08 to −0.01)	.01
Caffeine use	0.03 (0.00 to 0.06)	.09	0.04 (0.01 to 0.07)	.01	0.05 (0.01 to 0.08)	.009	0.01 (−0.02 to 0.05)	.48	−0.01 (−0.05 to 0.02)	.44
Physical activity	−0.04 (−0.07 to −0.01)	.01	−0.04 (−0.07 to 0.00)	.02	−0.05 (−0.08 to −0.01)	.004	−0.05 (−0.08 to −0.02)	.002	−0.01 (−0.05 to 0.02)	.41
Screen use	0.09 (0.06 to 0.13)	<.001	0.12 (0.09 to 0.15)	<.001	0.10 (0.07 to 0.13)	<.001	0.03 (0 to 0.07)	.05	0.03 (−0.01 to 0.06)	.09
2-Parent family	−0.07 (−0.10 to −0.04)	<.001	−0.08 (−0.11 to −0.05)	<.001	−0.06 (−0.10 to −0.03)	<.001	−0.01 (−0.04 to 0.03)	.78	0.02 (−0.01 to 0.06)	.19
Neighborhood deprivation score	0.14 (0.10 to 0.17)	<.001	0.14 (0.10 to 0.17)	<.001	0.13 (0.10 to 0.17)	<.001	0.09 (0.05 to 0.13)	<.001	0.05 (0.01 to 0.09)	.008
School start time	−0.01 (−0.04 to 0.02)	.50	−0.04 (−0.07 to −0.01)	.005	−0.04 (−0.07 to −0.01)	.009	−0.01 (−0.04 to 0.02)	.61	0.00 (−0.03 to 0.03)	.87

Abbreviations: BMI, body mass index; SES, socioeconomic status.

^a^ False discovery rate (significance level) was adjusted using the Benjamini-Hochberg procedure. Because log-transformed variability variables were used in the analysis, the unstandardized coefficients cannot be interpreted with substantive meaning.

Among racially and ethnically minoritized children, compared with Black children, Latinx children showed less variability in duration (β = −0.09; 95% CI, −0.15 to −0.04; *P* < .001), bedtime (β = −0.08; 95% CI, −0.13 to −0.03; *P* = .008), and efficiency (β = −0.18; 95% CI, −0.25 to −0.11; *P* < .001). Compared with Black children, multiracial children (β = −0.07; 95% CI, −0.12 to −0.01; *P* = .03) and Asian children (β = −0.07; 95% CI, −0.10 to −0.03; *P* < .001) also showed less variability in sleep efficiency. Moreover, multiracial children exhibited greater variability in sleep efficiency than Latinx children (β = 0.06; 95% CI, 0.02 to 0.10; *P* = .01).

#### Supplementary Analyses

When examining how racial and ethnic differences changed by introducing covariates 1 set at a time, 2 major patterns emerged (see eTable 6 and eTable 7 in Supplement 1). Compared with raw differences, Black children exhibited fewer sleep disparities after including contextual covariates, whereas Asian children exhibited more sleep disparities after introducing sociodemographic covariates. There was no association of race and ethnicity variables with weekday vs weekend sleep.

## Discussion

In this cross-sectional study using objective actigraphy sleep data from a national sample of children in the ABCD study, the current study investigated the mean levels and variability of multiple sleep dimensions across diverse racial and ethnic groups. Compared with their White peers, Asian, Black, Latinx, and multiracial children consistently exhibited shorter sleep duration and later bedtime, as well as greater variability in bedtime and risetime. While Black children exhibited the most profound disparities across a wide range of sleep dimensions than other groups, Asian and multiracial children also exhibited some disparities compared with their Latinx peers. These disparities were observed accounting for other potential sociodemographic, health, and contextual variations in children’s sleep. Findings were also consistent across weekdays and weekends.

This study replicated previous evidence on racial and ethnic disparities centering around mean sleep duration and bedtime.^16^ More importantly, the findings of this study suggest that these disparities need to be interpreted in the context of additional disparities related to sleep time variability. The observed differences can stem from multiple factors, none of which are attributed to the children themselves. Structural racism can expose racially and ethnically minoritized children to unfavorable environments (eg, neighborhoods) characterized by limited access to health care and high levels of crime, noise, and pollution.^46,47^ They may also be exposed to interpersonal discrimination on a daily basis,^48^ all contributing to changes in sleep from one day to another.^19,49^ Cultural differences in sleep-related beliefs and practices such as sleep schedule and arrangement may also explain the differential variability.^14^ Additionally, although cultural values such as familism are typically protective for racially and ethnically minoritized communities,^50^ demands from families can lead to less regular sleep.^29^ Emerging evidence has documented sleep variability as similarly or even more influential than sleep duration for children’s academic,^51^ psychosocial,^13,52^ physical,^36^ and brain development.^37,53^ The current findings suggest that racial and ethnic disparities in sleep time variability may be another important mechanism via which sleep contributes to inequalities in downstream developmental outcomes.

Turning to specific racial and ethnic groups, Black children exhibited a wider range of sleep disparities than their peers in both mean levels (duration and bedtime) and variability (duration, bedtime, risetime, and efficiency).^4,16^ Given the historical structural oppression faced by Black and African American communities,^54^ they may experience a constellation of disadvantages contributing to these pronounced sleep disparities. Supporting this idea, our supplementary analyses suggested that contextual influences like neighborhood deprivation and school start time may be especially important for Black children’s sleep. Accordingly, these factors should be targeted closely in practice. Moreover, this study provides insights into sleep disparities among Asian, Latinx, and multiracial children. In addition to differing from White children, Asian and multiracial children showed some disparities compared with Latinx children in sleep duration, efficiency, and efficiency variability. This finding underscores the need to investigate risk and resilience factors (eg, cultural values^55^) influencing sleep across diverse groups. Moreover, our supplementary analyses showed that that sociodemographic factors may mask Asian children’s sleep disparities, highlighting the need to further understand within-group heterogeneity among these children. Moreover, although Latinx children exhibited disparities compared with White children in some sleep dimensions, they had higher sleep efficiency. Future research is needed to understand whether this finding reflects better sleep quality or greater exhaustion.^56^

### Limitations

Due to limited group sizes, we could not examine some racial and ethnic groups (eg, single-race American Indian or Alaska Native or Pacific Islander children) or potential heterogeneity within Asian, Latinx, or multiracial children. Moreover, while the Fitbit model used in the ABCD study has demonstrated validity in assessing sleep^34,35^ and its association with developmental outcomes,^38,39,40^ it shares limitations with other research-based actigraphs, such as the accuracy in detecting wake epochs.^35^ The use of a proprietary algorithm also prevented corroboration with sleep diaries, a common practice for validating actigraphy data.^18^ Importantly, similar to previous research, the actigraphy subsample exhibited differences from the full ABCD sample (eg, children were more likely to be from higher SES and White families), possibly due to differential willingness and systemic barriers for participation in wearable-device based research.^57^ As such, actual sleep disparities may be even more pronounced than the patterns observed in this study. Additionally, although our sensitivity analyses, controlling for potential mediating factors (eg, racial discrimination or psychological distress), supported the robustness of the primary findings (results available upon request), future research is needed to investigate the developmental mechanisms underlying the observed sleep disparities.

## Conclusions

Despite these limitations, the current study makes a substantial contribution to the sleep health literature by investigating disparities in sleep variability across diverse racially and ethnically minoritized groups, including multiracial children, who are typically not included in such analyses. Understanding disparities in sleep variability, beyond just mean levels, is crucial for fully capturing the extent of these disparities and identifying areas for intervention. Our findings suggest that pediatric health clinicians should inquire about both mean levels and variability of sleep when working with children from diverse racial and ethnic groups,^58^ attending to cultural values and practices that influence sleep.^59^ Other efforts such as school programs and innovative mobile technologies seeking to promote pediatric sleep health should also develop interventions tailored to diverse groups.^60,61^ Addressing these multifaceted sleep disparities at multiple levels may be a promising avenue for promoting children’s health and development across diverse racial and ethnic backgrounds.

## Notes

Supplement 1. **eTable 1.** Bivariate Correlation Between Missingness in Sleep Data and Primary Study Variables and Covariates **eTable 2.** Validation Studies Comparing Fitbit Charge 2 and Widely Used Research Actigraphy Watches (Micro Motionlogger, Actiwatch) with Polysomnography (PSG) and Electroencephalography (EEG) Methods **eTable 3.** Bivariate Correlations Between Sleep Dimensions and Child Adjustment **eTable 4.** Measurement Details of Covariates **eTable 5.** Descriptive Statistics of Multiple Sleep Dimensions on Weekday and Weekends **eTable 6.** Covariates and Estimates for Racial and Ethnic Differences in Multiple Sleep Dimensions (Mean Levels) **eTable 7.** Covariates and Estimates for Racial and Ethnic Differences in Multiple Sleep Dimensions (Variability) **eTable 8.** Estimated Actual Racial and Ethnic Differences in Multiple Sleep Dimensions (Mean Levels)

Supplement 2.
Data Sharing Statement
