# Supplementary material for: Race, Ethnicity, and Sleep in US Children
Source: JAMA Netw Open. 2024 Dec 10;7(12):e2449861. doi: 10.1001/jamanetworkopen.2024.49861 (PMC11632548; doi:10.1001/jamanetworkopen.2024.49861)
Supplement: Supplement 1. — eTable 1. Bivariate Correlation Between Missingness in Sleep Data and Primary Study Variables and Covariates eTable 2. Validation Studies Comparing Fitbit Charge 2 and Widely Used Research Actigraphy Watches (Micro Motionlogger, Actiwatch) with Polysomnography (PSG) and Electroencephalography (EEG) Methods eTable 3. Bivariate Correlations Between Sleep Dimensions and Child Adjustment eTable 4. Measurement Details of Covariates eTable 5. Descriptive Statistics of Multiple Sleep Dimensions on Weekday and Weekends eTable 6. Covariates and Estimates for Racial and Ethnic Differences in Multiple Sleep Dimensions (Mean Levels) eTable 7. Covariates and Estimates for Racial and Ethnic Differences in Multiple Sleep Dimensions (Variability) eTable 8. Estimated Actual Racial and Ethnic Differences in Multiple Sleep Dimensions (Mean Levels) [file jamanetwopen-e2449861-s001.pdf]

Supplemental Online Content

Wang Y, Zhao Z, Yan J, et al. Race, ethnicity, and sleep in US children. *JAMA Netw Open*. 2024;7(12):e2449861.
doi:10.1001/jamanetworkopen.2024.49861

eTable 1. Bivariate Correlation Between Missingness in Sleep Data and Primary Study Variables and Covariates

eTable 2. Validation Studies Comparing Fitbit Charge 2 and Widely Used Research Actigraphy Watches (Micro Motionlogger, Actiwatch) with Polysomnography (PSG) and Electroencephalography (EEG) Methods

eTable 3. Bivariate Correlations Between Sleep Dimensions and Child Adjustment

eTable 4. Measurement Details of Covariates

eTable 5. Descriptive Statistics of Multiple Sleep Dimensions on Weekday and Weekends

eTable 6. Covariates and Estimates for Racial and Ethnic Differences in Multiple Sleep Dimensions (Mean Levels)

eTable 7. Covariates and Estimates for Racial and Ethnic Differences in Multiple Sleep Dimensions (Variability)

eTable 8. Estimated Actual Racial and Ethnic Differences in Multiple Sleep Dimensions (Mean Levels)

This supplemental material has been provided by the authors to give readers additional information about their work.

© 2024 Wang Y et al. *JAMA Network Open.*

eTable 1. Bivariate Correlation Between Missingness in Sleep Data and Primary Study Variables and Covariates

Variables	Missingness in sleep data
Race and ethnicity	
White	-0.11
Black	0.15
Latinx	0.04
Asian	-0.03
Multi-race	-0.02
Covariates	
Age	0.02
Female	-0.04
Sexual minority	0.02
Generational status	0.01
SES	-0.17
Two-parent family	-0.13
BMI	0.03
Caffeine use	0.04
Screen usage	0.09
Physical activity	-0.02
Neighborhood deprivation	0.10
School start time	-0.00

© 2024 Wang Y et al. *JAMA Network Open.*

eTable 2. Validation Studies Comparing Fitbit Charge 2 and Widely Used Research Actigraphy Watches (Micro Motionlogger, Actiwatch) with Polysomnography (PSG) and Electroencephalography (EEG) Methods

							Differences from ref. (PSG or EEG)		
Study	N	M_{age} ± SD	Days	Ref.	Sensitivity (%)	Specificity (%)	TST (min)	Efficiency (%)	SOL (min)
Fitbit Charge 2									
de Zambotti et al., 2018	44	17.3 ± 2.5	1	PSG	96	61	**-9**	-3.1	-8
Liang & Martell, 2018	25	24.8 ± 4.4	3	EEG	n.a.	n.a.	**-12.3**	1.5	-11.1
Haghayegh et al., 2020	33	27.2 ± 12.6	3	EEG	95	57	-17	4	-11
Summary across studies									
Range					95-96	57-61	9-17	1.5-4	8-11
Mean					95.50	59.00	-14.65	.80	-10.03
SD					.71	2.83	3.32	3.60	1.76
Micro Motionlogger									
Meltzer et al., 2012 [b]	115	8.8 ± 4.4	1	PSG	89/92	73/65	**-23.6/-2.4**	n.a.	**-4.4/-.4**
Rupp & Balkin, 2011	29	24.3 ± 5.4	1	PSG	96.2	63.6	**-4.7**	**1.2**	**1.1**
Eylon et al., 2023 [b]	21	26.2 ± 1.5	3	PSG	95/96	28/38	7.3/5.2	n.a.	n.a.
Haghayegh et al., 2020	33	27.2 ± 12.6	3	EEG	94	53	-16.1 [a]	-3.7 [a]	**9.7**
Summary across studies									
Range					89–96.2	28-73	-23.6-7.3	1.2	-10-1.1
Mean					93.70	53.43	-5.72	-1.25	2.95
SD					2.77	17.35	12.07	3.46	9.69
Actiwatch 2									
Toon et al., 2016	78	8.4 ± 4.0	1	PSG	93	63	17	4	**-21**
Meltzer et al., 2012 [c]	115	8.8 ± 4.4	1	PSG	97/93/89	54/69/77	-2.7/**25.9**/**-30.5**	n.a.	**-.4/5.0/-5.7**
Chee et al., 2021	53	15 - 19	8	PSG	91	86	**33.86**	n.a.	n.a.
Cook et al., 2017	21	26.5 ± 4.6	1	PSG	97	31	**40.6**	**7**	**-13.5**
Chinoy et al., 2021	34	28.1 ± 3.9	3	PSG	97	39	**23.9**	**-7.6**	**5**
Summary across studies									
Range					89–97	31–86	-30.5-43.0	-7.6-9.3	-21-5
Mean					93.88	57.12	14.52	1.66	-5.34
SD					2.94	18.74	26.53	7.33	9.59

Note. Bolded numbers indicate significant difference between actigraph watches and the reference. TST = Total sleep time. SOL = Sleep onset

© 2024 Wang Y et al. *JAMA Network Open.*

latency. *n.a.* = the sleep indicator was not available in the original study. [a] Estimates were calculated based on means from each assessment method as opposed to bias analyses. [b] Estimates before and after the slash were based on Sadeh and Cole-Kripke algorithms, respectively. [c] Estimates before, in between, and after the slashes were based on high, medium, and low sensitivity settings, respectively. [d] Estimates were based on number of awakenings instead of minutes. These estimates were not summarized across studies.

In addition to the validation studies reported here, a recent study (Reifman et al., 2024) reviewed 14 empirical studies of 18 unique sleep-tracker devices compared to PSG. This review suggested that, overall, these sleep-tracker devices overestimated sleep duration by 19 minutes (SD = 44 minutes). Of note, Fitbit Charge 2, the model used in the ABCD study, showed smaller sleep measure errors (i.e., 9 to 17 minutes for sleep duration). The review also conducted a simulation of these quantitative data for 30 consecutive nights, showing that sleep measurement errors from these devices compared to PSG was acceptable for about 80% of the time. In particular, the Fitbit Charge 2 yielded more satisfactory performance, having acceptable sleep measurement errors for 90% of the time (Reifman et al., 2024).

References

de Zambotti M, Goldstone A, Claudatos S, Colrain IM, Baker FC. A validation study of Fitbit Charge 2™ compared with polysomnography in adults. *Chronobiol. Int.* 2018;35(4):465-76.

Liang Z, Chapa Martell MA. Validity of consumer activity wristbands and wearable EEG for measuring overall sleep parameters and sleep structure in free-living conditions. *J. Healthc. Inform. Res.* 2018;2(1):152-78.

Haghayegh S, Khoshnevis S, Smolensky MH, Diller KR, Castriotta RJ. Performance assessment of new-generation Fitbit technology in deriving sleep parameters and stages. *Chronobiol. Int.* 2020;37(1):47-59.

Meltzer LJ, Walsh CM, Traylor J, Westin AM. Direct comparison of two new actigraphs and polysomnography in children and adolescents. *Sleep.* 2012;35(1):159-66.

Rupp TL, Balkin TJ. Comparison of Motionlogger Watch and Actiwatch actigraphs to polysomnography for sleep/wake estimation in healthy young adults. *Behav. Res. Methods.* 2011;43:1152-60.

Eylon G, Tikotzky L, Dinstein I. Performance evaluation of Fitbit Charge 3 and actigraphy vs. polysomnography: Sensitivity, specificity, and reliability across participants and nights. *Sleep Health.* 2023;9(4):407-16.

Toon E, Davey MJ, Hollis SL, Nixon GM, Horne RS, Biggs SN. Comparison of commercial wrist-based and smartphone accelerometers, actigraphy, and PSG in a clinical cohort of children and adolescents. *J. Clin. Sleep Med.* 2016;12(3):343-50.

Chee NI, Ghorbani S, Golkashani HA, Leong RL, Ong JL, Chee MW. Multi-night validation of a sleep tracking ring in adolescents compared with a research actigraph and polysomnography. *Nat. Sci. Sleep.* 2021:177-90.

Cook JD, Prairie ML, Plante DT. Utility of the Fitbit Flex to evaluate sleep in major depressive disorder: a comparison against polysomnography and wrist-worn actigraphy. *J. Affect. Disord*. 2017;217:299-305.

Chinoy ED, Cuellar JA, Huwa KE, Jameson JT, Watson CH, Bessman SC, Hirsch DA, Cooper AD, Drummond SP, Markwald RR. Performance of seven consumer sleep-tracking devices compared with polysomnography. *Sleep.* 2021;44(5):zsaa291.

Reifman J, Priezjev NV, Vital-Lopez FG. Can we rely on wearable sleep-tracker devices for fatigue management? *Sleep*. 2024;47:zsad288.

© 2024 Wang Y et al. *JAMA Network Open.*

eTable 3. Bivariate Correlations Between Sleep Dimensions and Child Adjustment

	Cognitive tasks				Self-reported outcomes					
	Inhibitory control & attention		Episodic memory		School involvement		Internalizing problems		Externalizing problems	
Mean levels										
Duration	0.08	<0.001	0.06	<0.001	0.09	<0.001	-0.09	<0.001	-0.09	<0.001
Bedtime	-0.07	<0.001	-0.08	<0.001	-0.12	<0.001	0.10	<0.001	0.08	<0.001
Risetime	0.08	<0.001	0.07	<0.001	0.02	0.26	0.003	0.003	-0.04	0.01
Efficiency	0.001	0.77	0.04	0.18	0.05	0.02	0.02	0.33	-0.01	0.59
Latency	-0.02	0.31	-0.03	0.005	-0.003	0.75	0.01	0.48	0.001	0.95
Variability										
Duration	-0.08	<0.001	-0.10	<0.001	-0.10	<0.001	0.11	<0.001	0.11	<0.001
Bedtime	-0.09	<0.001	-0.14	<0.001	-0.11	<0.001	0.13	<0.001	0.12	<0.001
Risetime	-0.09	<0.001	-0.11	<0.001	-0.09	<0.001	0.09	<0.001	0.10	<0.001
Efficiency	-0.07	<0.001	-0.08	<0.001	-0.05	<0.001	0.05	0.004	0.04	0.02
Latency	-0.01	0.18	-0.02	0.15	0.02	0.14	0.003	0.85	-0.005	0.77

Note. For cognitive tasks, inhibitory control & attention was assessed by the NIH Toolbox Flanker Inhibitory Control and Attention Test; episodic memory was assessed by the Picture Sequence Memory Test within the Modified NIH Toolbox Fluid Cognition Composite Test. For self-reported outcomes, school involvement was assessed by School Involvement subscale of the PhenX School Risk and Protective Factors; internalizing and externalizing problems were assessed by the Child Behavior Checklist.

© 2024 Wang Y et al. *JAMA Network Open.*

eTable 4. Measurement Details of Covariates

Measure	Source	Wave	Item	Response scale
Age	P	Y2	How old is the child?	N/A
Sex	P	BY	What sex was the child assigned at birth, on the original birth certificate?	0 = Male, 1 = Female
Sexual orientation	C	BY to Y2	Are you gay or bisexual?	0 = no, 1 = gay or bisexual at any wave from baseline to Y2<br><br>Of note, 4.9% (*n* = 478) of participants reported not understanding the question at all waves, in which case, they were coded as missing.
Generational status	P	BY	Was the child/biological father/biological mother born in the US?	0 = 1st or 2nd generation (adolescents or parents were foreign born), 1 = 3rd generation (both adolescents and parents were born in the United States)
Parental education	P	Y1	What is the highest grade or level of school you/your partner have completed or the highest degree you have received?	1 = Less than HS degree<br>2 = HS degree, GED or equivalent diploma<br>3 = Some college<br>4 = Associate degree: occupational or academic Program<br>5 = Bachelor degree<br>6 = Master degree<br>7 = Professional school degree or doctoral degree
Parental employment status	P	Y1	Are you/your partner working now, looking for work, retired, stay at home parent, a student, or something else?	0 = not working<br>1 = part-time working<br>2 = full-time working
Family economic hardship	P	Y1	*In the past 12 months, has there been a time when you and your immediate family experienced any of the following: …*	0 = No, 1 = Yes

© 2024 Wang Y et al. *JAMA Network Open.*

Measure	Source	Wave	Item	Response scale
			Needed food but couldn't afford to buy it or couldn't afford to go out to get it?<br>Were without telephone service because you could not afford it?<br>Didn't pay the full amount of the rent or mortgage because you could not afford it?<br>Were evicted from your home for not paying the rent or mortgage?<br>Had services turned off by the gas or electric company, or the oil company wouldn't deliver oil because payments were not made?<br>Had someone who needed to see a doctor or go to the hospital but didn't go because you could not afford it?<br>Had someone who needed a dentist but couldn't go because you could not afford it?	
Family structure	P	BY	If adolescent live with two parents	0 = No, 1 = Yes
BMI	A	Y2	BMI z scores based on youth weight and height assessed by the ABCD team	N/A
Caffeine intake	C	Y2	Typically, how many drinks of the following beverages did you have per week in the past 6 months? (i.e., Coffee, Espresso and espresso drinks with caffeine, Tea with caffeine, Soda with caffeine, Energy drinks)	N/A
Physical activity	C	Y2	During the past seven days, on how many days were you physically active for a total of at least 60 minutes per day?	0 = 0 days, 1 = 1 day, 2 = 2 days, 3 = 3 days, 4 = 4 days, 5 = 5 days, 6 = 6 days, 7 = 7 days
Bedtime screen usage	C	Y2	Watch or stream movies, videos, or TV shows<br>Play video games<br>Play music<br>Talk on the phone or text (If you do not own a phone, choose 0 nights)<br>Spend time online on social media (e.g. Facebook)<br>Spend time in chat rooms<br>Browse the Internet, Google-ing (not school related)<br>Use a computer/laptop for studying<br>Reading<br>In the past week, how often have you had phone calls, text messages or emails that wake you after trying to go to sleep?	1=0 nights in the past week; 2=1-2 nights; 3=3-4 nights; 4=5-7 nights
School start time	C	Y2	My usual school schedule starts at …	N/A
Neighborhood deprivation	G	BY	Scores of 17 items derived from the American Community Survey on education, employment, income, housing conditions, poverty rate, and infrastructure within a neighborhood	N/A

© 2024 Wang Y et al. *JAMA Network Open.*

Note. P = parent reported. C = child reported. A = assessment conducted by ABCD team. G = geocode data. BY = baseline year. Y1 = one-year follow-up. Y2 = two-year follow-up. R = Item was reverse coded. Socioeconomic status was created as a latent factor indicated by parental education (λ = .71), employment status (λ = .48), and family economic hardship (λ = -.39).

© 2024 Wang Y et al. *JAMA Network Open.*

eTable 5. Descriptive Statistics of Multiple Sleep Dimensions on Weekday and Weekends

	Descriptives		Bivariate correlations																	
			Mean levels								Variability									
	Mean	SD	Bed		Wake		Eff		Lat		Dur		Bed		Wake		Eff		Lat	
Mean levels																				
Duration (hours)	7.45	0.66	-0.40	<0.001	0.24	<0.001	0.13	<0.001	0.10	<0.001	-0.31	<0.001	-0.32	<0.001	-0.21	<0.001	-0.24	<0.001	0.12	<0.001
Bedtime (hours)	10.84	1.18	--		0.19	<0.001	0.02	0.13	-0.07	<0.001	0.37	<0.001	0.43	<0.001	0.49	<0.001	0.10	<0.001	-0.08	<0.001
Risetime (hours)	7.10	1.14			--		0.04	0.01	0.02	0.18	-0.11	<0.001	-0.11	<0.001	-0.28	<0.001	-0.12	<0.001	0.001	0.15
Efficiency (0-1)	0.87	0.02					--		0.02	0.26	-0.16	<0.001	-0.11	<0.001	-0.09	<0.001	-0.55	<0.001	0.04	0.66
Latency (minutes)	6.95	5.24							--		-0.01	0.56	-0.01	0.05	-0.01	0.60	0.03	0.13	0.83	<0.001
Variability																				
Duration (hours)	1.02	0.52									--		0.59	<0.001	0.62	<0.001	0.32	<0.001	0.003	0.86
Bedtime (hours)	1.07	0.78											--		0.52	<0.001	0.27	<0.001	-0.01	0.40
Risetime (hours)	1.44	1.84													--		0.21	<0.001	-0.01	0.56
Efficiency (0-1)	0.03	0.03															--		0.02	0.21
Latency (minutes)	8.22	7.90																	--	
Mean levels																				
Duration (hours)	7.53	0.78	-0.41	<0.001	0.19	<0.001	0.08	<0.001	0.12	<0.001	-0.23	<0.001	-0.25	<0.001	-0.20	<0.001	-0.12	<0.001	0.12	<0.001
Bedtime (hours)	11.17	1.36	--		0.11	0.79	0.01	0.09	-0.01	0.44	0.29	<0.001	0.37	<0.001	0.46	<0.001	0.07	<0.001	-0.02	0.45
Risetime (hours)	7.48	1.39			--		0.03	0.05	0.02	0.61	-0.10	<0.001	-0.05	0.03	-0.33	<0.001	-0.05	0.01	0.02	0.58
Efficiency (0-1)	0.87	0.03					--		0.04	0.11	-0.10	<0.001	-0.10	<0.001	-0.06	<0.001	-0.55	<0.001	0.02	0.67
Latency (minutes)	6.99	6.46							--		0.01	0.67	0.02	0.41	0.02	0.25	0.002	0.89	0.82	<0.001
Variability																				
Duration (hours)	1.07	0.66									--		0.42	<0.001	0.47	<0.001	0.19	<0.001	0.02	0.20
Bedtime (hours)	1.11	0.88											--		0.35	<0.001	0.17	<0.001	0.03	0.11
Risetime (hours)	1.54	2.04													--		0.11	<0.001	0.03	0.09
Efficiency (0-1)	0.03	0.03															--		0.03	0.12
Latency (minutes)	7.21	8.80																	--	

Note. Raw variability scores (i.e., SD of each participant's sleep data over three weeks) were used to estimate descriptive statistics. We then log-transformed of the raw variability scores when estimating correlations.

© 2024 Wang Y et al. *JAMA Network Open.*

eTable 6. *Covariates and Estimates for Racial and Ethnic Differences in Multiple Sleep Dimensions (Mean Levels)*

Predictors	No covariates		Sociodemo. covariates		Sociodemo. + health covariates		Sociodemo. + health + contextual covariates		Change pattern
	β (95% CI)	*p*	β (95% CI)	*p*	β (95% CI)	*p*	β (95% CI)	*p*	
**Sleep duration**									
White [Reference]									
Black	-0.27 (-0.31, -0.23)	< 0.001	-0.25 (-0.29, -0.21)	< 0.001	-0.21 (-0.25, -0.17)	< 0.001	-0.18 (-0.22, -0.14)	< 0.001	
Latinx	-0.09 (-0.12, -0.06)	< 0.001	-0.10 (-0.13, -0.06)	< 0.001	-0.07 (-0.10, -0.03)	< 0.001	-0.06 (-0.10, -0.03)	0.002	
Multirace	-0.07 (-0.10, -0.04)	< 0.001	-0.07 (-0.10, -0.04)	< 0.001	-0.06 (-0.09, -0.03)	< 0.001	-0.06 (-0.09, -0.03)	< 0.001	
Asian	-0.05 (-0.09, -0.02)	0.003	-0.07 (-0.11, -0.04)	< 0.001	-0.08 (-0.11, -0.04)	< 0.001	-0.08 (-0.11, -0.04)	< 0.001	
Black [Reference]									
Latinx	0.29 (0.23, 0.34)	< 0.001	0.29 (0.20, 0.32)	< 0.001	0.23 (0.18, 0.29)	< 0.001	0.19 (0.13, 0.25)	< 0.001	
Multirace	0.20 (0.15, 0.25)	< 0.001	0.20 (0.14, 0.23)	< 0.001	0.15 (0.11, 0.20)	< 0.001	0.12 (0.08, 0.17)	< 0.001	
Asian	**0.10 (0.06, 0.14)**	**< 0.001**	**0.10 (0.03, 0.11)**	**< 0.001**	**0.05 (0.006, 0.08)**	**0.03**	0.02 (-0.02, 0.06)	0.26	A
Latinx [Reference]									
Multirace	-0.006 (-0.04, 0.03)	0.72	-0.002 (-0.04, 0.03)	0.91	-0.01 (-0.05, 0.02)	0.49	-0.01 (-0.05, 0.02)	0.45	
Asian	-0.02 (-0.05, 0.02)	0.47	-0.04 (-0.07, -0.002)	0.05	**-0.05 (-0.08, -0.01)**	**0.007**	**-0.05 (-0.09, -0.02)**	**0.003**	B
Multirace [Reference]									
Asian	-0.01 (-0.05, 0.02)	0.59	-0.03 (-0.07, 0.001)	0.07	**-0.04 (-0.08, -0.006)**	**0.03**	**-0.05 (-0.08, -0.009)**	**0.02**	B
**Bedtime**									
White [Reference]									
Black	0.24 (0.20, 0.28)	< 0.001	0.21 (0.17, 0.25)	< 0.001	0.17 (0.13, 0.21)	< 0.001	0.13 (0.09, 0.17)	< 0.001	
Latinx	0.15 (0.12, 0.18)	< 0.001	0.13 (0.09, 0.17)	< 0.001	0.1 (0.07, 0.14)	< 0.001	0.1 (0.06, 0.13)	< 0.001	
Multirace	0.08 (0.05, 0.11)	< 0.001	0.07 (0.04, 0.10)	< 0.001	0.06 (0.03, 0.09)	< 0.001	0.05 (0.02, 0.09)	0.003	
Asian	0.04 (0.008, 0.07)	0.02	0.06 (0.03, 0.09)	< 0.001	0.06 (0.03, 0.09)	< 0.001	0.06 (0.03, 0.09)	< 0.001	
Black [Reference]									
Latinx	-0.19 (-0.25, -0.13)	< 0.001	-0.19 (-0.22, -0.10)	< 0.001	-0.14 (-0.20, -0.08)	< 0.001	-0.09 (-0.15, -0.03)	0.01	
Multirace	-0.17 (-0.22, -0.12)	< 0.001	-0.17 (-0.19, -0.09)	< 0.001	-0.11 (-0.16, -0.06)	< 0.001	-0.08 (-0.13, -0.03)	0.004	
Asian	**-0.1 (-0.14, -0.06)**	**< 0.001**	**-0.10 (-0.10, -0.03)**	**0.001**	**-0.04 (-0.08, -0.003)**	**0.05**	-0.01 (-0.05, 0.03)	0.52	A
Latinx [Reference]									
Multirace	-0.03 (-0.07, 0.009)	0.17	-0.02 (-0.06, 0.01)	0.29	-0.02 (-0.05, 0.02)	0.41	-0.02 (-0.05, 0.02)	0.41	
Asian	-0.02 (-0.05, 0.008)	0.17	0.004 (-0.03, 0.04)	0.77	0.01 (-0.02, 0.05)	0.40	0.02 (-0.008, 0.05)	0.19	
Multirace [Reference]									
Asian	-0.007 (-0.04, 0.03)	0.70	0.02 (-0.02, 0.05)	0.35	0.02 (-0.01, 0.06)	0.22	0.03 (-0.002, 0.06)	0.09	
**Risetime**									
White [Reference]									
Black	**-0.08 (-0.12, -0.04)**	**< 0.001**	**-0.06 (-0.10, -0.01)**	**0.05**	-0.04 (-0.08, 0.005)	0.22	-0.02 (-0.07, 0.02)	0.68	C
Latinx	0.004 (-0.03, 0.04)	0.82	0.02 (-0.02, 0.06)	0.45	0.04 (-0.001, 0.08)	0.27	0.04 (0.004, 0.08)	0.16	
Multirace	-0.02 (-0.05, 0.02)	0.48	-0.01 (-0.05, 0.02)	0.67	-0.007 (-0.04, 0.03)	0.86	-0.01 (-0.04, 0.03)	0.89	

© 2024 Wang Y et al. *JAMA Network Open.*

Asian	0.009 (-0.02, 0.04)	0.73	-0.003 (-0.04, 0.03)	0.87	-0.003 (-0.04, 0.03)	0.94	-0.002 (-0.04, 0.03)	0.99	
Black [Reference]									
Latinx	**0.12 (0.05, 0.18)**	**< 0.001**	**0.12 (0.04, 0.17)**	**0.02**	**0.09 (0.03, 0.16)**	**0.05**	0.07 (0.008, 0.14)	0.28	A
Multirace	**0.06 (0.01, 0.11)**	**0.05**	0.06 (-0.007, 0.10)	0.31	0.03 (-0.02, 0.08)	0.51	0.02 (-0.04, 0.07)	0.95	D
Asian	**0.05 (0.01, 0.09)**	**0.03**	0.05 (-0.01, 0.07)	0.33	0.02 (-0.02, 0.06)	0.58	0.01 (-0.03, 0.05)	0.92	D
Latinx [Reference]									
Multirace	-0.02 (-0.06, 0.02)	0.56	-0.03 (-0.07, 0.01)	0.37	-0.04 (-0.07, 0.004)	0.25	-0.04 (-0.08, 0.001)	0.19	
Asian	0.008 (-0.03, 0.04)	0.74	-0.01 (-0.05, 0.02)	0.65	-0.02 (-0.06, 0.02)	0.49	-0.02 (-0.06, 0.02)	0.74	
Multirace [Reference]									
Asian	0.02 (-0.02, 0.06)	0.44	0.004 (-0.03, 0.04)	0.92	<0.001 (-0.04, 0.04)	0.98	0.001 (-0.04, 0.04)	0.95	
**Sleep efficiency**									
White [Reference]									
Black	0.005 (-0.03, 0.04)	0.85	0.009 (-0.03, 0.05)	0.70	0.01 (-0.03, 0.05)	0.63	0.02 (-0.02, 0.06)	0.50	
Latinx	0.06 (0.03, 0.10)	< 0.001	0.07 (0.03, 0.10)	0.005	0.07 (0.03, 0.10)	0.005	0.07 (0.03, 0.11)	0.005	
Multirace	-0.03 (-0.06, 0.007)	0.25	-0.03 (-0.06, 0.008)	0.26	-0.03 (-0.06, 0.008)	0.24	-0.02 (-0.06, 0.009)	0.31	
Asian	-0.01 (-0.05, 0.02)	0.66	-0.02 (-0.05, 0.01)	0.38	-0.02 (-0.05, 0.01)	0.38	-0.02 (-0.05, 0.01)	0.31	
Black [Reference]									
Latinx	0.06 (-0.001, 0.11)	0.13	0.06 (-0.007, 0.11)	0.21	0.05 (-0.009, 0.11)	0.25	0.04 (-0.02, 0.10)	0.30	
Multirace	-0.03 (-0.08, 0.02)	0.31	-0.03 (-0.08, 0.01)	0.25	-0.04 (-0.09, 0.01)	0.28	-0.04 (-0.09, 0.008)	0.27	
Asian	-0.02 (-0.05, 0.02)	0.53	-0.02 (-0.06, 0.01)	0.27	-0.03 (-0.06, 0.01)	0.25	-0.03 (-0.07, 0.007)	0.72	
Latinx [Reference]									
Multirace	-0.07 (-0.11, -0.04)	< 0.001	-0.07 (-0.11, -0.04)	< 0.001	-0.07 (-0.11, -0.03)	< 0.001	-0.07 (-0.11, -0.03)	< 0.001	
Asian	-0.04 (-0.07, -0.006)	0.06	**-0.05 (-0.08, -0.01)**	**0.02**	**-0.05 (-0.08, -0.01)**	**0.02**	**-0.05 (-0.08, -0.02)**	**0.01**	B
Multirace [Reference]									
Asian	0.001 (-0.03, 0.04)	0.78	-0.006 (-0.04, 0.03)	0.76	-0.006 (-0.04, 0.03)	0.74	-0.01 (-0.04, 0.03)	0.72	
**Sleep latency**									
White [Reference]									
Black	0.05 (0.02, 0.09)	0.06	0.05 (0.01, 0.09)	0.10	0.05 (0.008, 0.09)	0.17	0.04 (0.001, 0.08)	0.23	
Latinx	0.01 (-0.02, 0.04)	0.55	0.003 (-0.04, 0.04)	0.87	0.006 (-0.04, 0.05)	0.78	0.01 (-0.04, 0.05)	0.25	
Multirace	0.03 (-0.008, 0.06)	0.35	0.02 (-0.01, 0.06)	0.36	0.02 (-0.01, 0.06)	0.40	0.02 (-0.01, 0.06)	0.48	
Asian	-0.008 (-0.04, 0.02)	0.57	-0.02 (-0.04, 0.01)	0.36	-0.02 (-0.05, 0.01)	0.41	-0.01 (-0.04, 0.02)	0.40	
Black [Reference]									
Latinx	-0.06 (-0.12, -0.005)	0.11	-0.06 (-0.12, -0.009)	0.08	-0.06 (-0.12, -0.003)	0.13	-0.05 (-0.11, 0.005)	0.25	
Multirace	-0.03 (-0.07, 0.02)	0.45	-0.03 (-0.07, 0.02)	0.45	-0.03 (-0.07, 0.02)	0.38	-0.02 (-0.07, 0.03)	0.50	
Asian	-0.04 (-0.07, -0.004)	0.15	-0.04 (-0.08, -0.008)	0.08	-0.04 (-0.08, -0.007)	0.17	-0.04 (-0.07, -0.002)	0.58	
Latinx [Reference]									
Multirace	0.02 (-0.02, 0.05)	0.52	0.02 (-0.02, 0.06)	0.37	0.02 (-0.02, 0.06)	0.40	0.02 (-0.02, 0.06)	0.50	
Asian	-0.01 (-0.04, 0.02)	0.50	-0.02 (-0.05, 0.01)	0.40	-0.02 (-0.05, 0.01)	0.38	-0.02 (-0.05, 0.01)	0.58	
Multirace [Reference]									
Asian	-0.02 (-0.05, 0.01)	0.35	-0.03 (-0.06, 0.005)	0.24	-0.03 (-0.06, 0.004)	0.22	-0.03 (-0.06, 0.006)	0.95	

© 2024 Wang Y et al. *JAMA Network Open.*

Note. Changes in significance levels from the raw difference model to the full model are bolded. Change pattern A = Black children's sleep disparities became not significant after accounting for contextual covariates. B = Asian children's sleep disparities became significant or marginally significant after accounting for sociodemographic covariates. C = Black children's sleep disparities became not significant after accounting for health covariates. D = Black children's sleep disparities became not significant after accounting for sociodemographic variables.

© 2024 Wang Y et al. *JAMA Network Open.*

eTable 7. Covariates and Estimates for Racial and Ethnic Differences in Multiple Sleep Dimensions (Variability)

Variables	No covariates		Sociodemo. covariates		Sociodemo. + health covariates		Sociodemo. + health + contextual covariates		Change pattern
	β (95% CI)	*p*	*β* (95% CI)	*p*	*β* (95% CI)	*p*	*β* (95% CI)	*p*	
**Sleep duration**									
White [Reference]									
Black	0.22 (0.19, 0.25)	< 0.001	0.16 (0.13, 0.20)	< 0.001	0.13 (0.10, 0.17)	< 0.001	0.09 (0.05, 0.12)	< 0.001	
Latinx	**0.09 (0.06, 0.12)**	**< 0.001**	**0.06 (0.02, 0.10)**	**0.003**	0.04 (0.001, 0.08)	0.08	0.03 (-0.007, 0.07)	0.19	D
Multirace	0.07 (0.03, 0.10)	< 0.001	0.06 (0.02, 0.09)	0.002	0.05 (0.01, 0.08)	0.01	0.04 (0.009, 0.07)	0.04	
Asian	-0.005 (-0.04, 0.03)	0.84	0.03 (-0.01, 0.06)	0.23	0.03 (-0.009, 0.06)	0.22	0.03 (-0.005, 0.06)	0.18	
Black [Reference]									
Latinx	-0.22 (-0.27, -0.17)	< 0.001	-0.17 (-0.22, -0.12)	< 0.001	-0.15 (-0.20, -0.10)	< 0.001	-0.09 (-0.15, -0.04)	< 0.001	
Multirace	**-0.15 (-0.20, -0.11)**	**< 0.001**	**-0.11 (-0.15, -0.07)**	**< 0.001**	**-0.09 (-0.13, -0.05)**	**< 0.001**	-0.05 (-0.09, -0.006)	0.06	A
Asian	**-0.13 (-0.17, -0.09)**	**< 0.001**	**-0.07 (-0.11, -0.03)**	**< 0.001**	**-0.05 (-0.09, -0.01)**	**0.02**	-0.02 (-0.06, 0.02)	0.38	A
Latinx [Reference]									
Multirace	0.002 (-0.03, 0.04)	0.90	0.01 (-0.02, 0.05)	0.65	0.02 (-0.02, 0.05)	0.39	0.02 (-0.02, 0.05)	0.42	
Asian	**-0.04 (-0.07, -0.006)**	**0.03**	0.001 (-0.03, 0.04)	0.97	0.01 (-0.03, 0.04)	0.65	0.02 (-0.02, 0.05)	0.36	B
Multirace [Reference]									
Asian	**-0.04 (-0.08, -0.005)**	**0.03**	-0.006 (-0.04, 0.03)	0.83	-0.001 (-0.04, 0.04)	0.97	0.01 (-0.03, 0.04)	0.71	B
**Bedtime**									
White [Reference]									
Black	0.25 (0.22, 0.28)	< 0.001	0.2 (0.17, 0.23)	< 0.001	0.16 (0.13, 0.20)	< 0.001	0.11 (0.08, 0.15)	< 0.001	
Latinx	0.14 (0.11, 0.17)	< 0.001	0.12 (0.08, 0.15)	< 0.001	0.09 (0.05, 0.12)	< 0.001	0.08 (0.05, 0.12)	< 0.001	
Multirace	0.11 (0.07, 0.14)	< 0.001	0.1 (0.07, 0.13)	< 0.001	0.09 (0.06, 0.12)	< 0.001	0.08 (0.05, 0.11)	< 0.001	
Asian	-0.002 (-0.03, 0.03)	0.89	**0.03 (0.004, 0.07)**	**0.04**	**0.03 (0.005, 0.06)**	**0.03**	**0.04 (0.01, 0.07)**	**0.02**	B
Black [Reference]									
Latinx	-0.22 (-0.27, -0.17)	< 0.001	-0.17 (-0.22, -0.12)	< 0.001	-0.14 (-0.19, -0.09)	< 0.001	-0.08 (-0.13, -0.03)	0.008	
Multirace	**-0.15 (-0.19, -0.10)**	**< 0.001**	**-0.1 (-0.15, -0.06)**	**< 0.001**	**-0.08 (-0.12, -0.03)**	**0.002**	-0.03 (-0.08, 0.01)	0.21	A
Asian	**-0.15 (-0.18, -0.11)**	**< 0.001**	**-0.08 (-0.11, -0.04)**	**< 0.001**	**-0.06 (-0.09, -0.02)**	**0.002**	-0.03 (-0.06, 0.009)	0.24	A
Latinx [Reference]									
Multirace	0.009 (-0.03, 0.05)	0.69	0.02 (-0.02, 0.05)	0.40	0.03 (0.06, 0.14)	0.22	0.03 (-0.01, 0.06)	0.19	
Asian	**-0.06 (-0.09, -0.03)**	**< 0.001**	-0.01 (-0.04, 0.02)	0.46	-0.001 (-0.01, 0.06)	0.95	0.01 (-0.02, 0.04)	0.66	B
Multirace [Reference]									
Asian	**-0.06 (-0.10, -0.03)**	**< 0.001**	-0.02 (-0.05, 0.01)	0.27	-0.02 (-0.05, 0.02)	0.42	-0.01 (-0.04, 0.02)	0.71	B
**Risetime**									
White [Reference]									
Black	0.22 (0.18, 0.25)	< 0.001	0.16 (0.12, 0.19)	< 0.001	0.12 (0.09, 0.16)	< 0.001	0.08 (0.04, 0.12)	< 0.001	
Latinx	0.14 (0.11, 0.17)	< 0.001	0.09 (0.06, 0.13)	< 0.001	0.07 (0.03, 0.10)	0.003	0.06 (0.02, 0.10)	0.01	
Multirace	**0.07 (0.03, 0.10)**	**< 0.001**	**0.05 (0.02, 0.08)**	**0.004**	**0.04 (0.008, 0.07)**	**0.02**	0.04 (0.004, 0.07)	0.07	E

© 2024 Wang Y et al. *JAMA Network Open.*

Asian	0.01 (-0.02, 0.04)	0.48	**0.04 (0.009, 0.07)**	**0.02**	**0.04 (0.01, 0.07)**	**0.02**	**0.04 (0.01, 0.07)**	**0.01**	B
Black [Reference]									
Latinx	**-0.17 (-0.23, -0.12)**	**< 0.001**	**-0.13 (-0.18, -0.07)**	**< 0.001**	**-0.11 (-0.16, -0.05)**	**< 0.001**	-0.05 (-0.11, 0.005)	0.12	A
Multirace	**-0.16 (-0.20, -0.11)**	**< 0.001**	**-0.11 (-0.15, -0.06)**	**< 0.001**	**-0.08 (-0.13, -0.04)**	**< 0.001**	-0.04 (-0.09, 0.002)	0.12	A
Asian	**-0.11 (-0.15, -0.08)**	**< 0.001**	**-0.05 (-0.08, -0.01)**	**0.008**	-0.03 (-0.06, 0.005)	0.13	-0.001 (-0.04, 0.03)	0.96	C
Latinx [Reference]									
Multirace	-0.03 (-0.07, 0.004)	0.10	-0.02 (-0.05, 0.02)	0.50	-0.007 (-0.04, 0.03)	0.70	-0.01 (-0.04, 0.03)	0.80	
Asian	-0.04 (-0.08, -0.01)	0.006	**0.002 (-0.03, 0.03)**	**0.91**	**0.01 (-0.02, 0.04)**	**0.45**	**0.02 (-0.01, 0.05)**	**0.24**	B
Multirace [Reference]									
Asian	-0.03 (-0.06, 0.007)	0.13	0.01 (-0.02, 0.04)	0.59	0.02 (-0.02, 0.05)	0.38	0.02 (-0.008, 0.06)	0.21	
**Sleep efficiency**									
White [Reference]									
Black	0.17 (0.13, 0.21)	< 0.001	0.15 (0.11, 0.19)	< 0.001	0.13 (0.09, 0.18)	< 0.001	0.11 (0.07, 0.16)	< 0.001	
Latinx	-0.01 (-0.04, 0.02)	0.51	-0.006 (-0.05, 0.03)	0.74	-0.02 (-0.06, 0.02)	< 0.001	-0.02 (-0.06, 0.02)	0.34	
Multirace	0.05 (0.01, 0.08)	0.01	0.05 (0.01, 0.09)	0.01	0.05 (0.01, 0.08)	0.002	0.04 (0.008, 0.08)	0.03	
Asian	-0.03 (-0.06, -0.002)	0.05	-0.008 (-0.04, 0.02)	0.71	-0.008 (-0.04, 0.02)	0.67	-0.004 (-0.03, 0.02)	0.77	
Black [Reference]									
Latinx	-0.25 (-0.31, -0.18)	< 0.001	-0.21 (-0.28, -0.15)	< 0.001	-0.21 (-0.27, -0.14)	< 0.001	-0.02 (-0.08, 0.03)	< 0.001	
Multirace	-0.12 (-0.17, -0.07)	< 0.001	-0.1 (-0.15, -0.05)	< 0.001	-0.09 (-0.14, -0.04)	0.002	-0.002 (-0.05, 0.05)	0.03	
Asian	-0.12 (-0.16, -0.09)	< 0.001	-0.09 (-0.13, -0.06)	< 0.001	-0.08 (-0.12, -0.05)	< 0.001	-0.02 (-0.06, 0.02)	< 0.001	
Latinx [Reference]									
Multirace	0.06 (0.02, 0.10)	0.008	0.05 (0.02, 0.09)	0.01	0.06 (0.02, 0.10)	0.005	0.06 (0.02, 0.10)	0.01	
Asian	-0.02 (-0.05, 0.004)	0.10	-0.006 (-0.03, 0.02)	0.78	-0.001 (-0.03, 0.03)	0.97	0.01 (-0.02, 0.03)	0.83	
Multirace [Reference]									
Asian	**-0.06 (-0.09, -0.02)**	**0.002**	**-0.04 (-0.07, -0.004)**	**0.04**	**-0.03 (-0.07, -0.001)**	**0.04**	-0.03 (-0.06, 0.003)	0.11	E
**Sleep latency**									
White [Reference]									
Black	0.03 (-0.001, 0.07)	0.56	0.03 (-0.006, 0.06)	0.99	0.03 (-0.009, 0.06)	0.99	0.02 (-0.02, 0.06)	0.78	
Latinx	0.01 (-0.02, 0.04)	0.74	0.003 (-0.04, 0.04)	0.89	0.004 (-0.04, 0.05)	0.83	0.003 (-0.04, 0.04)	0.97	
Multirace	0.02 (-0.01, 0.05)	0.57	0.02 (-0.02, 0.05)	0.54	0.02 (-0.02, 0.05)	0.58	0.02 (-0.02, 0.05)	0.99	
Asian	-0.006 (-0.04, 0.03)	0.72	-0.01 (-0.05, 0.02)	0.65	-0.01 (-0.05, 0.02)	0.60	-0.01 (-0.04, 0.02)	0.74	
Black [Reference]									
Latinx	-0.04 (-0.09, 0.02)	0.93	-0.04 (-0.09, 0.02)	0.60	-0.03 (-0.09, 0.02)	0.60	-0.18 (-0.25, -0.11)	0.82	
Multirace	-0.01 (-0.06, 0.03)	0.64	-0.01 (-0.06, 0.04)	0.73	-0.009 (-0.06, 0.04)	0.77	-0.07 (-0.12, -0.01)	0.93	
Asian	-0.03 (-0.06, 0.01)	0.93	-0.03 (-0.07, 0.01)	0.79	-0.03 (-0.07, 0.01)	0.82	-0.07 (-0.10, -0.03)	0.98	
Latinx [Reference]									
Multirace	0.01 (-0.02, 0.05)	0.84	0.02 (-0.02, 0.06)	0.65	0.02 (-0.02, 0.05)	0.64	0.02 (-0.02, 0.05)	0.72	
Asian	-0.01 (-0.04, 0.02)	0.69	-0.01 (-0.05, 0.02)	0.69	-0.01 (-0.05, 0.02)	0.71	-0.01 (-0.05, 0.02)	0.78	
Multirace [Reference]									
Asian	-0.02 (-0.05, 0.02)	0.69	-0.02 (-0.06, 0.02)	0.62	-0.02 (-0.06, 0.02)	0.78	-0.02 (-0.06, 0.02)	0.99	

© 2024 Wang Y et al. *JAMA Network Open.*

Note. Changes in significance levels from the raw difference model to the full model are bolded. Change pattern A = Black children's sleep disparities became not significant after accounting for contextual covariates. B = Asian children's disparities became significant or their advantages became non-significant after accounting for sociodemographic covariates. C = Black children's sleep disparities became not significant after accounting for health covariates. D = Latinx children's sleep disparities became not significant after accounting for health covariates. E = Multiracial chilren's sleep disparities became not significant after accounting for contextual covariates.

© 2024 Wang Y et al. *JAMA Network Open.*

eTable 8. Estimated Actual Racial and Ethnic Differences in Multiple Sleep Dimensions (Mean Levels)

	Duration (minutes)	Bedtime (minutes)	Risetime (minutes)	Efficiency (%)	Latency (minutes)
White [Reference]					
Black	~24	~33			
Latinx	~6	~18		~0.3	
Multirace	~8	~14			
Asian	~18	~28			
Black [Reference]					
Latinx	~18	~16	~13		
Multirace	~17	~20			
Asian					
Latinx [Reference]					
Multirace				~0.5	
Asian	~13			~0.6	
Multirace [Reference]					
Asian	~11				

Note. Racial and ethnic differences were estimated using unstandardized coefficients. Blank cells indicated non-significant racial and ethnic differences.

© 2024 Wang Y et al. *JAMA Network Open.*
