# Supplementary material for: Race, Ethnicity, and Sleep in US Children
Source: JAMA Netw Open. 2024 Dec 10;7(12):e2449861. doi: 10.1001/jamanetworkopen.2024.49861 (PMC11632548; doi:10.1001/jamanetworkopen.2024.49861)
Supplement: Supplement 2. — Data Sharing Statement [file jamanetwopen-e2449861-s002.pdf]

Data Sharing Statement

Wang. Race, Ethnicity, and Sleep in US Children. *JAMA Netw Open*. Published December 10, 2024. doi:10.1001/jamanetworkopen.2024.49861

Data
Data available: No

Additional Information
Explanation for why data not available: The study was a secondary data analysis of the ABCD Study. Data access can be obtained through the ABCD Study.
